# Prevalence of infectious diseases in preterm infants: a 2-year follow-up from the Japan Environment and Children’s Study

**DOI:** 10.1038/s41598-022-26748-0

**Published:** 2022-12-28

**Authors:** Kentaro Tamura, Kenta Matsumura, Akiko Tsuchida, Taketoshi Yoshida, Hidekuni Inadera, The Japan Environment and Children’s Study (JECS) Group, Michihiro Kamijima, Shin Yamazakii, Yukihiro Ohya, Reiko Kishi, Nobuo Yaegashi, Koichi Hashimoto, Chisato Mori, Shuichi Ito, Zentaro Yamagata, Takeo Nakayama, Tomotaka Sobue, Masayuki Shima, Hiroshige Nakamura, Narufumi Suganuma, Koichi Kusuhara, Takahiko Katoh

**Affiliations:** 1 Division of Neonatology, Maternal and Perinatal Center, Toyama University Hospital, 2630 Sugitani, Toyama, 930-0194 Japan; 2 Department of Public Health, Faculty of Medicine, University of Toyama, 2630 Sugitani, Toyama, 930-0194 Japan; 3 Toyama Regional Center for JECS, University of Toyama, Toyama, Japan; 4 Graduate School of Medical Sciences Department of Occupational and Environmental Health, Nagoya City University, 1 Kawasumi, Mizuho-Cho, Mizuho-Ku, Nagoya, Aichi 467-8601 Japan; 5 National Institute for Environmental Studies, Tsukuba, Japan; 6 National Center for Child Health and Development, Tokyo, Japan; 7 Hokkaido University, Sapporo, Japan; 8 Tohoku University, Sendai, Japan; 9 Fukushima Medical University, Fukushima, Japan; 10 Chiba University, Chiba, Japan; 11 Yokohama City University, Yokohama, Japan; 12 University of Yamanashi, Chuo, Japan; 13 Kyoto University, Kyoto, Japan; 14 Osaka University, Suita, Japan; 15 Hyogo Medical University, Nishinomiya, Japan; 16 Tottori University, Yonago, Japan; 17 Kochi University, Nankoku, Japan; 18 University of Occupational and Environmental Health, Kitakyushu, Japan; 19 Kumamoto University, Kumamoto, Japan

**Keywords:** Infectious diseases, Preterm birth, Paediatric research

## Abstract

Evidence regarding the long-term risk of infections in preterm infants is lacking. In this study, we examined whether preterm infants developed various common childhood infections more frequently than full-term infants by the age of 2 years by analyzing data from a questionnaire completed by 67,282 mother–toddler pairs in a nationwide birth cohort study. Of the target population, 2885 (4.3%) were born prematurely. After covariate adjustment for maternal and children factors, lower respiratory tract infections appeared more frequent in preterm than in full-term infants at both 1 and 2 years (adjusted odds ratio [aOR] 1.21, 95% confidence interval [CI] 1.05–1.41, and aOR 1.27, 95% CI 1.11–1.46, respectively). However, there was no significant difference in the frequencies of lower respiratory tract infection between preterm and full-term infants after Palivizumab administration. The risk of other common infections, such as in the upper respiratory tract infection, otitis media, urinary tract infection, gastroenteritis, herpangina, hand-foot-and-mouth disease, chickenpox, influenza virus, and adenovirus infections, was not higher in preterm than in full-term infants after covariates adjustment for maternal and children factors. These findings suggest Palivizumab prophylaxis could reduce the frequencies of lower respiratory tract infection in preterm to the same level as in full-term infants.

## Introduction

In Western and developed countries, approximately 5–12% of all pregnancies result in preterm birth and this rate has not decreased over the past two to three decades^1^. In Japan, the preterm birth rate has remained at 5.4–5.7% since 2000^2^. Although survival has significantly improved for extremely preterm infants, severe bacterial infectious diseases, such as sepsis, bacterial meningitis, and pneumonia, remain a cause of neonatal death and long-term complications during neonatal intensive care unit (NICU) admission^3^. Infections can also contribute to a lower quality of life for preterm infants after hospitalization from the NICU to home; infection-related deaths and hospitalizations increased significantly in extremely preterm infants^4,5^ who show persistently higher rates of infection-related hospital admissions until 18 years of age^6,7^. However, differences in the frequency of various common childhood infections between preterm and full-term infants have not yet been fully investigated. Several studies have reported that the frequency of viral respiratory infections in preterm infants is comparable to that of full-term infants after hospitalization^8,9^. Respiratory syncytial virus (RSV) is considered the most common cause of lower respiratory tract infection in infants. Similar to the American Academy of Pediatrics indications, Palivizumab, a humanized monoclonal antibody, has been used widely in Japan since 2002 against RSV F glycoprotein^10^. However, population-based data investigating the effect of Palivizumab on RSV infections after its widespread use are still lacking.

In this research, we compared the frequency of the different common childhood infections between preterm and full-term infants by the age of 2 years, after hospitalization, using data extracted from the Japan Environment and Children’s Study (JECS). Furthermore, we assessed the effects of Palivizumab on RSV-associated infectious diseases on post-hospitalized preterm infants.

## Results

The characteristics of the participants in preterm and full-term births are presented in Table 1; out of 67,282 mothers, 2885 (4.3%) delivered prematurely, whereas 64,397 (95.7%) had a full-term delivery. There were significant differences in maternal age at delivery, pre-pregnancy body mass index (BMI), history of maternal allergy, physical activity, alcohol intake, maternal active and passive smoking history, marital status, Cesarean section, child sex, major anomaly, feeding methods during the first month after birth, and anti-RSV vaccine received before 1 and 2 years old. In contrast, no significant differences were detected regarding parity, energy intake during pregnancy, annual household income, highest education level, employment during early pregnancy, birth season, and attending a childcare facility.

**Table 1:** Characteristics of the participants.

	Preterm birth		Full term birth		*p*
	(n = 2885)		(n = 64,397)		
	n	(%)	n	(%)	
**Age at early pregnancy, year**					< 0.0001
< 25	202	(7.0)	5546	(8.6)	
25 to < 30	716	(24.8)	18,681	(29.0)	
30 to < 35	1019	(35.3)	23,518	(36.5)	
≥ 35	948	(32.9)	16,652	(25.9)	
**Pre-pregnancy body mass index, kg/m**^**2**^					< 0.0001
< 18.5	535	(18.5)	10,160	(15.8)	
18.5 to < 25	1966	(68.2)	47,946	(74.5)	
≥ 25	384	(13.3)	6291	(9.8)	
**Parity**					0.2226
Primipara	1223	(42.4)	28,040	(43.5)	
Multipara	1662	(57.6)	36,357	(56.5)	
**History of maternal allergy**					0.0105
No	1496	(51.9)	31,825	(49.4)	
Yes	1389	(48.2)	32,572	(50.6)	
**Physical activity corresponding to over 10-minute walk/day**					< 0.0001
No	865	(30.0)	15,161	(23.5)	
Yes	2020	(70.0)	49,236	(76.5)	
**Quartile of energy intake during pregnancy**					0.1215
Q1 (≤ 1300 kcal/day)	717	(24.9)	15,362	(23.9)	
Q2 (1300–1614 kcal/day)	686	(23.8)	16,576	(25.7)	
Q3 (1615–2009 kcal/day)	747	(25.9)	16,380	(25.4)	
Q4 (≥ 2010 kcal/day)	735	(25.5)	16,079	(25.0)	
**Alcohol intake**					0.0007
Never	1057	(36.6)	21,525	(33.4)	
Former	1765	(61.2)	41,117	(63.9)	
Current	63	(2.2)	1755	(2.7)	
**Active smoking history**					0.0046
Never	1708	(59.2)	38,872	(60.4)	
Former	1046	(36.2)	23,327	(36.2)	
Current	131	(4.5)	2198	(3.4)	
**Passive smoking history**					0.0010
Never	1857	(64.4)	41,592	(64.6)	
1–6 days /week	654	(22.7)	15,753	(24.5)	
Everyday	374	(13.0)	7052	(11.0)	
**Annual household income, million JPY**					0.2177
< 4	1149	(39.8)	24,718	(38.4)	
4 to < 6	965	(33.5)	21,671	(33.7)	
≥ 6	771	(26.7)	18,008	(28.0)	
**Highest education level, year**					0.4720
< 12	972	(33.7)	21,071	(32.7)	
12 to < 16	1246	(43.2)	27,944	(43.4)	
≥ 16	667	(23.1)	15,382	(23.9)	
**Employed during early pregnancy**					0.1652
No	1343	(46.5)	29,131	(45.2)	
Yes	1542	(53.5)	35,266	(54.8)	
**Marital status**					0.0001
Married	2773	(96.1)	62,167	(96.5)	
Single	75	(2.6)	1823	(2.8)	
Divorced or widowed	37	(1.3)	407	(0.6)	
**Caesarean section**					< 0.0001
No	1658	(57.5)	53,163	(82.6)	
Yes	1227	(42.5)	11,234	(17.4)	
**Child sex**					< 0.0001
Boy	1699	(58.9)	32,766	(50.9)	
Girl	1186	(41.1)	31,631	(49.1)	
**Major anomaly**					< 0.0001
No	2718	(94.2)	63,086	(98.0)	
Yes	167	(5.8)	1311	(2.0)	
**Birth season**					0.1057
Spring (March–May)	684	(23.7)	15,054	(23.4)	
Summer (June–August)	804	(27.9)	17,109	(26.6)	
Autumn (September–November)	736	(25.5)	17,723	(27.5)	
Winter (December–February)	661	(22.9)	14,511	(22.5)	
**Feeding methods during the first month after birth**					< 0.0001
Breastfeeding only	891	(30.9)	28,038	(43.5)	
Mixed feeding	1927	(66.8)	35,621	(55.3)	
Infant formula only	67	(2.3)	738	(1.2)	
**Attending a childcare facility at 6 months of age**					0.1511
No	2714	(94.1)	60,144	(93.4)	
Yes	171	(5.9)	4253	(6.6)	
**Palivizumab administration**					< 0.0001
Yes	1169	(41.7)	504	(0.8)	
No or unsure	1635	(58.3)	61,775	(99.2)	

Table 2 shows crude and adjusted odds ratios (aOR) with 95% confidence intervals (CI) of the association between preterm birth and infections at 1 and 2 years of age; in the crude model, preterm birth was associated significantly with increased risks of lower respiratory tract and urinary tract infections at 1 year, and lower respiratory tract infection at 2 years in the offspring compared with full-term infants (OR 1.24, 95% CI 1.08–1.44, *p* = 0.0028; OR 1.57, 95% CI 1.10–2.24, *p* = 0.0131; OR 1.28, 95% CI 1.12–1.46, *p* = 0.0004, respectively). In contrast, preterm birth was associated with decreased risks of otitis media and exanthema subitum at 1 year of age compared with full-term birth (OR 0.87, 95% CI 0.76–0.98, *p* = 0.0225; OR 0.86, 95% CI 0.78–0.95, *p* = 0.0024, respectively). However, after covariates adjustment by adding maternal factors (Model 1), as well as children factors such as feeding methods and attending a childcare facility (Model 2), only lower respiratory tract infections were more frequent in preterm than full-term infants at both years 1 and 2 (aOR 1.21, 95% CI 1.05–1.41, *p* = 0.0102; aOR 1.27, 95% CI 1.11–1.46, *p* = 0.0007, respectively), whereas the urinary tract infections did not show any significant difference between preterm and full-term infants at 2 years of age. In contrast, preterm birth was associated inversely with otitis media and exanthema subitem risk at 1 year of age compared with full-term birth in both Models 1 and 2, similar to the results of crude OR (Model 1 [otitis media; aOR 0.85, 95% CI 0.75–0.97, *p* = 0.0134, exanthema subitum; aOR 0.89, 95% CI 0.80–0.98, *p* = 0.0170] and Model 2 [otitis media; aOR 0.87, 95% CI 0.77–0.99, *p* = 0.0336, exanthema subitum; aOR 0.90, 95% CI 0.81–0.995, *p* = 0.0397]).

**Table 2:** Associations between preterm birth and infections at 1 year and 2 years of age.

	Cases				Crude		Multivariate Model 1^a^		Multivariate Model 2^b^	
	Preterm (n = 2885)		Full term (n = 64,397)							
	n	(%)	n	(%)	OR [95% CI]	*p*	OR [95% CI]	*p*	OR [95% CI]	*p*
**1 year of age**										
Upper respiratory tract infection	671	(23.3)	15,004	(23.3)	1.00 [0.91–1.09]	0.9594	0.99 [0.91–1.09]	0.8965	1.00 [0.91–1.09]	0.9075
Lower respiratory tract infection	213	(7.4)	3878	(6.0)	**1.24 [1.08–1.44]**	**0.0028**	**1.21 [1.05–1.41]**	**0.0095**	**1.21 [1.05–1.41]**	**0.0102**
Otitis media	288	(10.0)	7315	(11.4)	**0.87 [0.76–0.98]**	**0.0225**	**0.85 [0.75–0.97]**	**0.0134**	**0.87 [0.77–0.99]**	**0.0336**
Urinary tract infection	33	(1.1)	472	(0.7)	**1.57 [1.10–2.24]**	**0.0131**	1.43 [1.00–2.06]	0.0504	1.43 [1.00–2.05]	0.0533
Gastroenteritis	267	(9.3)	5839	(9.1)	1.02 [0.90–1.16]	0.7293	1.00 [0.88–1.14]	0.9917	0.99 [0.87–1.13]	0.8731
Exanthema subitum	505	(17.5)	12,753	(19.8)	**0.86 [0.78–0.95]**	**0.0024**	**0.89 [0.80–0.98]**	**0.0170**	**0.90 [0.81–1.00]**	**0.0397**
Herpangina	109	(3.8)	2421	(3.8)	1.01 [0.83–1.22]	0.9588	1.04 [0.85–1.27]	0.6947	1.05 [0.86–1.28]	0.6351
Hand-foot-and-mouth disease	163	(5.6)	4217	(6.5)	0.86 [0.73–1.00]	0.0559	0.88 [0.75–1.03]	0.1176	0.89 [0.75–1.05]	0.1547
Chickenpox	99	(3.4)	2333	(3.6)	0.95 [0.77–1.16]	0.5903	0.95 [0.77–1.17]	0.6324	0.97 [0.78–1.19]	0.7423
Influenza virus infection	167	(5.8)	3372	(5.2)	1.11 [0.95–1.31]	0.1937	1.10 [0.93–1.29]	0.2585	1.11 [0.94–1.30]	0.2339
RS virus infection	279	(9.7)	6152	(9.6)	1.01 [0.89–1.15]	0.8329	0.99 [0.87–1.13]	0.9260	1.00 [0.88–1.14]	0.9927
Adenovirus infection	80	(2.8)	1789	(2.8)	1.00 [0.80–1.25]	0.9870	0.98 [0.78–1.24]	0.8686	0.99 [0.78–1.25]	0.9119
**2 years of age**										
Upper respiratory tract infection	682	(23.6)	14,231	(22.1)	1.09 [1.00–1.19]	0.0513	**1.10 [1.00–1.20]**	**0.0429**	1.09 [1.00–1.20]	0.0515
Lower respiratory tract infection	240	(8.3)	4271	(6.6)	**1.28 [1.12–1.46]**	**0.0004**	**1.28 [1.11–1.47]**	**0.0005**	**1.27 [1.11–1.46]**	**0.0007**
Otitis media	505	(17.5)	10,755	(16.7)	1.06 [0.96–1.17]	0.2582	1.05 [0.95–1.16]	0.3612	1.06 [0.96–1.17]	0.2757
Urinary tract infection	11	(0.4)	205	(0.3)	1.20 [0.65–2.20]	0.5590	1.04 [0.56–1.92]	0.9100	1.03 [0.56–1.91]	0.9216
Gastroenteritis	378	(13.1)	7830	(12.2)	1.09 [0.98–1.22]	0.1300	1.09 [0.98–1.22]	0.1280	1.10 [0.98–1.23]	0.1136
Exanthema subitum	456	(15.8)	9683	(15.0)	1.06 [0.96–1.18]	0.2584	1.09 [0.98–1.21]	0.1001	1.08 [0.98–1.20]	0.1278
Herpangina	184	(6.4)	4286	(6.7)	0.96 [0.82–1.11]	0.5648	0.98 [0.84–1.14]	0.7866	0.98 [0.84–1.14]	0.7698
Hand-foot-and-mouth disease	420	(14.6)	9690	(15.0)	0.96 [0.87–1.07]	0.4773	0.99 [0.89–1.11]	0.9013	1.00 [0.89–1.11]	0.9241
Chickenpox	138	(4.8)	2847	(4.4)	1.09 [0.91–1.29]	0.3552	1.11 [0.93–1.33]	0.2359	1.13 [0.95–1.35]	0.1776
Influenza virus infection	265	(9.2)	5549	(8.6)	1.07 [0.94–1.22]	0.2877	1.06 [0.93–1.21]	0.3889	1.06 [0.93–1.21]	0.3875
RS virus infection	252	(8.7)	4948	(7.7)	**1.15 [1.01–1.31]**	**0.0387**	1.14 [1.00–1.31]	0.0566	1.14 [1.00–1.31]	0.0531
Adenovirus infection	156	(5.4)	3306	(5.1)	1.06 [0.90–1.25]	0.5154	1.06 [0.90–1.26]	0.4662	1.08 [0.91–1.27]	0.3924

Boldface indicates significance.

^a^ Adjusted for maternal age, pre-pregnancy body mass index, parity, history of maternal allergy, history of any physical disease, marital status, employed, highest education level, annual household income, alcohol intake, smoking history, physical activity.

^b^Adjusted for all the covariates in Model 1 and feeding methods and attending a childcare facility.

n = 67,282.

To determine the mediating effects of RSV and lower respiratory tract infections in preterm birth, we included Palivizumab as a covariate (Table 3); after adjusting Model 2 and having received Palivizumab (Model 3), there was no significant difference in the frequency of lower respiratory tract infection between preterm and full-term infants (1 year; aORs 1.11, 95% CI 0.92–1.32, *p* = 0.2731, 2 years; aORs 1.10, 95% CI 0.93–1.30, *p* = 0.2859). The frequency of RSV infections in preterm and full-term infants did not differ in Models 3, with no significant difference.

**Table 3:** Mediating effect of Palivizumab on RSV and lower respiratory tract infections in preterm infants.

	Birth	Crude		Multivariate Model 2^a^		Multivariate Model 3^b^	
		OR [95% CI]	*p*	OR [95% CI]	*p*	OR [95% CI]	*p*
**1 year of age**							
Lower respiratory tract infection	Preterm	**1.25 [1.08–1.44]**	**0.0031**	**1.22 [1.05–1.41]**	**0.0105**	1.11 [0.92–1.32]	0.2731
	Full-term	Reference		Reference		Reference	
RS virus infection	Preterm	0.99 [0.87–1.12]	0.8321	0.97 [0.85–1.11]	0.6552	1.11 [0.96–1.30]	0.1626
	Full-term	Reference		Reference		Reference	
**2 years of age**							
Lower respiratory tract infection	Preterm	**1.27 [1.11–1.46]**	**0.0006**	**1.27 [1.11–1.47]**	**0.0007**	1.10 [0.93–1.30]	0.2859
	Full-term	Reference		Reference		Reference	
RS virus infection	Preterm	**1.16 [1.02–1.33]**	**0.0255**	**1.16 [1.01–1.33]**	**0.0346**	1.16 [0.99–1.37]	0.0607
	Full-term	Reference		Reference		Reference	

Boldface indicates significance.

^a^Adjusted for maternal age, pre-pregnancy body mass index, parity, history of maternal allergy, history of any physical disease, marital status, employed, highest education level, annual household income, alcohol intake, smoking history, physical activity, feeding methods, and attending a childcare facility.

^b^Adjusted for all the covariates in Model 2 and having received Palivizumab, a monoclonal antibody against the RSV.

n = 62,731.

## Discussion

This study found that preterm infants developed lower respiratory tract infections more frequently than full-term infants by 1 and 2 years of age, however, the risks were comparable after receiving Palivizumab. The occurrence of other common childhood infections in preterm infants after hospitalization, however, was not significantly higher than that in full-term infants.

In Japan, the preterm birth rate was 5.6% in 2015^2^, although it counted for 4.6% in our current study, in which approximately 80% of the preterm infants were late preterm (34–36 weeks of gestational age), similar to the provided data on preterm births in Japan^2^. Although the no response or missing data rate for extremely preterm infants might be higher, we assume that our study represents the current situation of preterm and full-term birth infants in Japan. Several studies have reported that preterm infants might have poor protection against infections owing to an overactive or poorly responsive innate immune system, suppressed chemokine production, and immature neutrophil function^11,12^. Owing to these immunological mechanisms, the vaccine response in preterm infants could be inferior to that in full-term infants^11,13,14^. In addition, serum immunoglobulin G (IgG) levels are lower in preterm infants because less IgG is transferred through the placenta^15^. Consequently, we believe that healthcare providers should be able to segregate the infections according to their higher risk in compromising the health of preterm infants, not only during NICU admission, but also after discharge.

In our study, only the frequency of lower respiratory tract infection remained higher in both age groups in preterm infants, after covariates adjustment for maternal and children factors. Concerning the mechanism, preterm infants might be more susceptible to lower respiratory tract inflammation owing to either bronchopulmonary dysplasia or airway hyper-responsiveness. In this context, several studies have reported that preterm infants have a higher incidence of lower respiratory tract infections, which might contribute to more treatments requiring hospitalization and higher medical costs compared with full-term infants^5,7,13,16^. However, these studies did not show any association between the incidence of lower respiratory tract infection and Palivizumab prophylaxis. RSV is considered the most common cause of lower respiratory tract infection in children < 1 year of age^17^. Although the efficacy of Palivizumab in reducing RSV-related hospitalizations was well established^18–21^, our study suggests that Palivizumab administration reduced the incidence of lower respiratory tract infection in preterm infants to the same level as in full-term infants. Recently, some studies have reported that Nirsevimab, an Fc-modified monoclonal antibody against RSV with an extended half-life, was effective in preventing RSV-associated lower respiratory tract infection in healthy preterm and full-term infants^22,23^. Once Nirsevimab is approved for use, further studies on its effectiveness will be needed.

Our data show that the incidence of otitis media and exanthema subitum by 1-year of age was lower in preterm than in full-term infants, even after adjustment for maternal and postnatal factors (Table 2). This is because preterm infants spent more days in the hospital, therefore, it is expected that they spent less time at home before 1 year. In this context, Caserta et al. showed that the daily risk of acquiring a respiratory viral infection in preterm infants in the NICU is significantly lower than in full-term infants living in a normal community^8^. Consequently, preterm infants who spent less time in the community before the age of one might have less opportunity to develop otitis media and exanthema subitum.

In our data, there was no difference in the incidence of other infections between preterm and full-term infants up to 1 and 2 years of age even after adjustment for confounding factors (Table 2). Van den Berg showed that transplacental IgG transport for measles, mumps, rubella, and varicella zoster were significantly lower in preterm infants than in full-term infants^24^. Other studies revealed that preterm infants mounted antibody responses that were similar to those of full-term infants after vaccination^25^. Although there is limited information about the associations between other viral infections and preterm infants, hospitalizations due to the influenza virus infection (H1N1) were similar between preterm and full-term infants^9^. Studies on adenovirus, herpangina, and hand-foot-mouth disease are still lacking. However, although gastroenteritis is considered the second most common reason of postneonatal hospitalization by infection for preterm infants^26^, in our cohort study, there was no notable difference concerning this infection between preterm and full-term infants.

The strengths of our study are as follows; (1) large size of the sample (> 60,000 mother–child pairs), (2) we recruited this sample from 15 regional centers in Japan within the past decade. Therefore, we trust that our records are illustrative of the recent Japanese mother–child pairs, and (3) we added several variables that probably impact the development of infectious diseases in children, including not only maternal characteristics, socioeconomic, and smoking history, but also maternal parity, feeding methods, and attending a childcare facility. Finally, to our knowledge, our study is the first report to present the occurrence of various common infections in preterm infants compared with full-term infants after discharge from a neonatal care facility.

Several limitations also need to be considered; (1) the questionnaire regarding infections depended on the mother’s memory, and data were not obtained from medical records. In addition, the questionnaire missed information on multiple infections during the period, infection severity, or whether hospitalization was needed; and (2) the no response or missing data rate might have been higher in more preterm infants because questions about infants start after birth.

In conclusion, our data show that preterm infants are at a higher risk of lower respiratory tract infection than full-term infants by 1 and 2 years of age, however, the risk was comparable after adjustment for Palivizumab administration. Nevertheless, the occurrence of other common childhood infections in preterm infants after discharge from a neonatal care facility was not significantly higher than for full-term infants.

## Methods

### Study design and participants

**Figure 1:**
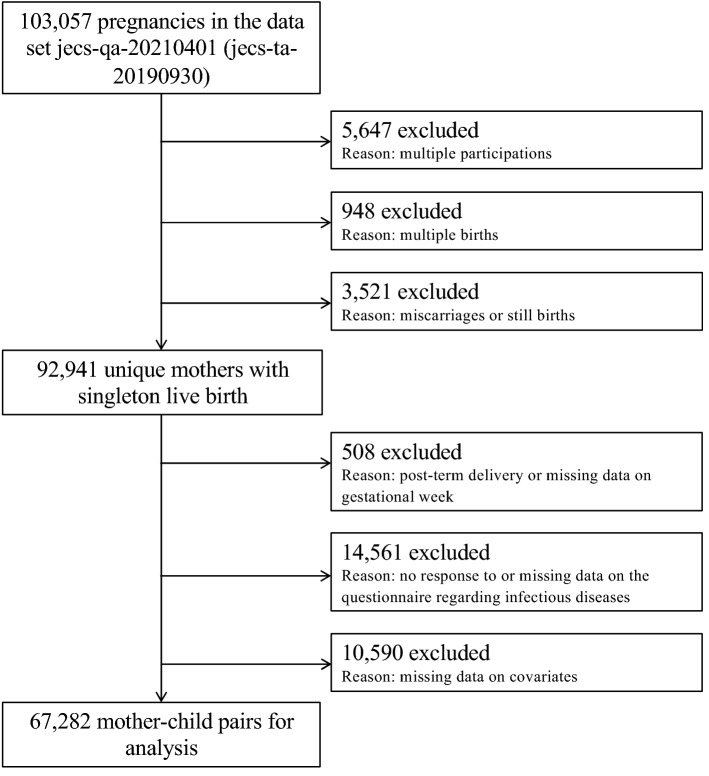
Participant flow diagram. Flow diagram of the recruitment and exclusion process for participants.

Features of JECS have been published comprehensively in terms of model and design elsewhere^27–29^. In brief, the JECS is a cohort study about birth, funded by the Japan government to reach all of Japan, and it includes many elements that influence the wellbeing and growth of children. The participants were enrolled in person in 15 Regional Centres throughout Japan between January 2011 and March 2014. The current study analyzed the set of data referenced by jecs-qa-20210401 (jecs-ta-20190930), which was available in April 2021. Data from 103,057 pregnancies until 3 years postnatal were included, from which we omitted records with inappropriate data for analysis, such as those with numerous participation, multiple births, miscarriages/still births, post-term delivery, missing data on gestational week, no response to or missing data on the questionnaire regarding infectious diseases, or missing data on covariates. Therefore, 67,282 mother–child pairs remained for the last examination (Fig. 1).

The authors declared that ethical standards complied with all processes defining this research from relevant national and institutional committees on studies comprising participation from human as per the declaration of Helsinki. The JECS protocol was reviewed and approved by the Ministry of the Environment’s Institutional Review Board on Epidemiological Studies (no. 100910001), the Ethics Committees of all participating institutions, and the Committee of Ethics at Toyama University (no. R2022055). Finally, we obtained written informed consent from all participants.

### Data collection

First, we gave the questionnaire for every participant autonomously to be auto-completed at trimesters one, two or three, and at 1 month, 6 months, 1 year, 1.5 year and 2 years after delivery. The questionnaire had many inquiries regarding demographic factors, socioeconomic status, lifestyle, occupation, medical history, physical and mental health, and housing conditions. We transcribed perinatal medical records from each cooperating health care provider, including gestational age and infant physical examinations of the infant at birth and at 1 month of age.

### Outcome measures and covariates

The frequency of infection development in infants is regarded as the first outcome. Infections included upper and lower respiratory tract infections, otitis media, urinary tract infection, gastroenteritis, exanthema subitum, herpangina, hand-foot-mouth disease, chickenpox, influenza virus, RSV infection, and adenovirus infections. We assessed the data when the infants were at 1 and 2 years of age, through the following question: “Has your child been diagnosed with any of these infections by a physician since the last survey until the present time?”.

The covariates involved in the analysis were maternal age, prepregnancy BMI, parity, history of maternal allergy, history of any physical disease, marital status, maternal employment, maternal highest education level, annual household income, maternal alcohol intake, maternal smoking history, maternal physical activity, feeding methods, attending a childcare facility at 6 months of age, and receiving Palivizumab prophylaxis.

### Statistical analysis

The participants were classified into preterm (< 37 weeks) and full-term (37 till < 42 weeks) birth groups according to the gestational weeks for the statistical analysis. We used t-test and chi-square to evaluate the significant difference between the two groups. We conducted multivariable logistic regression analysis to identify the relationship between preterm birth and the frequency of infant infections by the age of 1 and 2 years.

We used the compulsory entry method to comprise covariates in the multivariable analysis. We adjusted the regression in Model 1 for maternal age, pre-pregnancy BMI, parity, history of maternal allergy, history of any physical disease, marital status, employment, highest education level, annual household income, total energy intake assessed using Food Frequency Questionnaire^30^, alcohol intake, smoking history, and physical activity^31,32^. Next, we adjusted Model 2 by adding to the first model, feeding methods, and attending a childcare facility. Finally, we adjusted Model 3 by adding the inquiry regarding Palivizumab administration to the Model 2.

We present the results as crude and aORs with 95% CI, we set the significance at *p* < 0.05, and used SAS 9.4 (SAS Institute Inc., Cary, North Carolina) for statistical analyses.

## Data Availability

Data are unsuitable for public deposition due to ethical restrictions and the legal framework of Japan. It is prohibited by the Act on the Protection of Personal Information (Act No. 57 of 30 May 2003, amendment on 9 September 2015) to publicly deposit data containing personal information. Ethical Guidelines for Medical and Health Research Involving Human Subjects enforced by the Japan Ministry of Education, Culture, Sports, Science, and Technology and the Ministry of Health, Labour, and Welfare also restrict the open sharing of the epidemiological data. All inquiries about access to data should be sent to: jecs-en@nies.go.jp. The person responsible for handling inquiries sent to this e-mail address is Dr Shoji F. Nakayama, JECS Programme Office, National Institute for Environmental Studies.
